# Determinant roles of dendritic cell-expressed Notch Delta-like and Jagged ligands on anti-tumor T cell immunity

**DOI:** 10.1186/s40425-019-0566-4

**Published:** 2019-04-02

**Authors:** Elena E. Tchekneva, Mounika U.L. Goruganthu, Roman V. Uzhachenko, Portia L. Thomas, Anneliese Antonucci, Irina Chekneva, Michael Koenig, Longzhu Piao, Anwari Akhter, Maria Teresa P. de Aquino, Parvathi Ranganathan, Nicholas Long, Thomas Magliery, Anna Valujskikh, Jason V. Evans, Rajeswara R. Arasada, Pierre P. Massion, David P. Carbone, Anil Shanker, Mikhail M. Dikov

**Affiliations:** 1 Division of Medical Oncology, Department of Internal Medicine, The Ohio State University Wexner Medical Center and The James Comprehensive Cancer Center, 460 W 12th Ave, 484 BRT, Columbus, OH 43210 USA; 2 Department of Biochemistry, Cancer Biology, Neuroscience and Pharmacology, Meharry Medical College School of Medicine, 2005 Harold D. West Basic Sciences Building, 1023 21st Ave N, Nashville, 37208 TN USA; 3 Department of Microbiology, Immunology and Physiology, Meharry Medical College School of Medicine, Nashville, USA; 4 School of Graduate Studies and Research, Meharry Medical College, Nashville, TN USA; 5 Sechenov First Moscow State Medical University, Moscow, Russia; 6 Division of Hematology, Department of Internal Medicine, The Ohio State University Wexner Medical Center, Columbus, OH USA; 7 Department of Chemistry and Biochemistry, The Ohio State University, Columbus, OH USA; 8 Department of Inflammation and Immunity, Cleveland Clinic, Cleveland, OH USA; 9 Department of Pathology, West Virginia University, Morgantown, WV USA; 10 Department of Medicine, Vanderbilt University, Nashville, TN USA; 11 Host–Tumor Interactions Research Program, Vanderbilt-Ingram Comprehensive Cancer Center, Vanderbilt University, Nashville, TN USA; 12 Vanderbilt Institute for Infection, Immunology and Inflammation, Vanderbilt University, Nashville, TN USA

**Keywords:** Delta-like notch ligands, Jagged, Notch receptors, Lung carcinoma, Tumor infiltrating immune cells, Heart allograft rejection, Dendritic cells, CD8 T-cells, Regulatory T-cells, Cancer immunotherapy

## Abstract

**Background:**

Notch intercellular communication instructs tissue-specific T-cell development and function. In this study, we explored the roles of dendritic cell (DC)-expressed Notch ligands in the regulation of T-cell effector function.

**Methods:**

We generated mice with CD11c lineage-specific deletion of Notch Delta-like ligand *(Dll)1* and Jagged *(Jag)2.* Using these genetically-ablated mice and engineered pharmacological Notch ligand constructs, the roles of various Delta-like and Jagged ligands in the regulation of T-cell-mediated immunity were investigated. We assessed tumor growth, mouse survival, cytokine production, immunophenotyping of myeloid and lymphoid populations infiltrating the tumors, expression of checkpoint molecules and T-cell function in the experimental settings of murine lung and pancreatic tumors and cardiac allograft rejection. Correlative studies were also performed for the expression of NOTCH ligands, NOTCH receptors and PD-1 on various subsets of myeloid and lymphoid cells in tumor-infiltrating immune cells analyzed from primary human lung cancers.

**Results:**

Mice with CD11c lineage-specific deletion of Notch ligand gene *Dll1*, but not *Jag2*, exhibited accelerated growth of lung and pancreatic tumors concomitant with decreased antigen-specific CD8^+^T-cell functions and effector-memory (Tem) differentiation. Increased IL-4 but decreased IFN-γ production and elevated populations of T-regulatory and myeloid-derived suppressor cells were observed in *Dll1*-ablated mice. Multivalent clustered DLL1-triggered Notch signaling overcame DC *Dll1* deficiency and improved anti-tumor T-cell responses, whereas the pharmacological interference by monomeric soluble DLL1 construct suppressed the rejection of mouse tumors and cardiac allograft. Moreover, monomeric soluble JAG1 treatment reduced T-regulatory cells and improved anti-tumor immune responses by decreasing the expression of PD-1 on CD8^+^Tem cells. A significant correlation was observed between DC-expressed Jagged and Delta-like ligands with Tem-expressed PD-1 and Notch receptors, respectively, in human lung tumor-infiltrates.

**Conclusion:**

Our data show the importance of specific expression of Notch ligands on DCs in the regulation of T-cell effector function. Thus, strategies incorporating selectively engineered Notch ligands could provide a novel approach of therapeutics for modulating immunity in various immunosuppressive conditions including cancer.

**Electronic supplementary material:**

The online version of this article (10.1186/s40425-019-0566-4) contains supplementary material, which is available to authorized users.

## Background

Signals delivered to naïve T-cells by antigen-presenting cells (APCs) along with a specific cytokine milieu play key roles in regulation of CD4^+^ and CD8^+^ T-cell differentiation. Accumulating evidence suggests that engagement of Notch ligands presented by APCs with Notch receptors on T-cells are important regulators of T-cell differentiation [1]. The mammalian Notch family presents an intricate intercellular communication system that includes four known transmembrane receptors, Notch1–4, and five cell membrane-bound Notch ligands, Delta-like (DLL)1, DLL3, DLL4, Jagged (Jag)1 and Jag2. Notch regulates a variety of processes in cellular development and differentiation in a dose and context-dependent manner [2]. Notch target genes belong to various functional classes that act as transcriptional repressors to downregulate expression of tissue-specific transcriptional activators, or regulators of cell cycle and apoptosis [3, 4].

In the immune system, Notch provides instructive signals for priming CD4^+^T-cells and governing the differentiation of T helper (Th), follicular Th, and regulatory T (Treg) cells [5–11]. Notch has been shown to promote Th1 differentiation by upregulating *T-bet* and *Ifn-γ* expression [12]. It can also transactivate Th2-promoting genes *Il4* and *Gata3* [6]. Notch ligand-specific signaling can alter Th1 or Th2 differentiation with different ligands supporting distinct polarization of Th cells [13–16]. Most gain-of-function studies indicate that Delta-like ligands promote CD4^+^T-cell commitment to Th1 type [17, 18]. Although controversy exists, studies support that Jagged ligands induce Th2-promoting Notch signaling [17, 19]. Notch also regulates *Il17* and *RORγt* gene promoters to influence Th17 differentiation [8]. In addition to guiding Th1, Th2 and Th17 differentiation, expression of Jagged ligands by APCs or hematopoietic progenitors can favor generation of suppressive T-cells in vitro or Treg cells in vivo [20–22]. Systemic blockade of Jag1 and 2 with Jagged ligand-specific antibodies overcame tumor-induced T-cell tolerance, indicating the involvement of these ligands in T-cell suppression [23]. Expression of Delta-like ligands, but not Jagged, in hematopoietic compartments was altered by tumor-derived factors to cause tumor-induced immunosuppression [20, 24, 25]. An alternative hypothesis posits that interaction of DLL4 expressed by dendritic cells (DCs) and Notch1 on T-cells may fine-tune sensitivity, magnitude and quality of the CD4^+^T-cell response by promoting metabolic reprogramming, rather than by specifying lineage choice following the initial exposure to the antigen [21]. It is known that a transient pulse with high levels of Delta-like ligands can induce Hes1 expression for a duration that is sufficient to induce a binary cell fate switch in T-cell or natural killer cell differentiation [22]. Both Notch1 and Notch2 have been identified as key players in anti-tumor T-cell immunity including induction of tumor-specific cytotoxic T lymphocytes (CTL) and memory T-cells [21, 23, 26]. Studies also indicate that Notch regulates effector cytokine production by CD8^+^T-cells [5, 27, 28].

It is, however, unclear what specific roles different Notch ligands play in modulating T-cell responses. In this study, we used genetic and pharmacological approaches to investigate the roles of various Delta-like and Jagged ligands in the regulation of T-cell-mediated immunity in mouse models of lung and pancreatic tumors and cardiac allograft rejection. We found that DC-expressed DLL1, but not Jag2, is indispensable for the induction of antigen-specific responses and generation of effector and memory T-cells. In human lung tumor infiltrates, we noted a significant correlation between Jag1 or Jag2-expressing DCs with the PD-1-expressing CD8^+^T effector-memory (Tem) cells. In contrast, expression of DLL1 or DLL4 in DC was positively correlated with the expression of Notch receptors on tumor-infiltrating Tem cells. In mice lacking DLL1 in CD11c^+^ cells, a Notch-activating clustered DLL1 construct could compensate for the genetic deficiency of DLL1 on DCs. Moreover, treatment with soluble JAG1 resulted in the decreased differentiation of Treg cells, a decreased expression of PD-1 molecules on CD8^+^Tem cells and improved anti-tumor responses. These data emphasize the importance of specific expression of Notch ligands on DCs by revealing their distinct roles in the regulation of T-cell immunity, and suggest opportunities for modulating immune outcomes using engineered Notch ligand constructs.

## Results

### Deletion of *Dll1* but not *Jag2* in dendritic cells accelerates tumor growth and decreases host survival

**Fig. 1:**
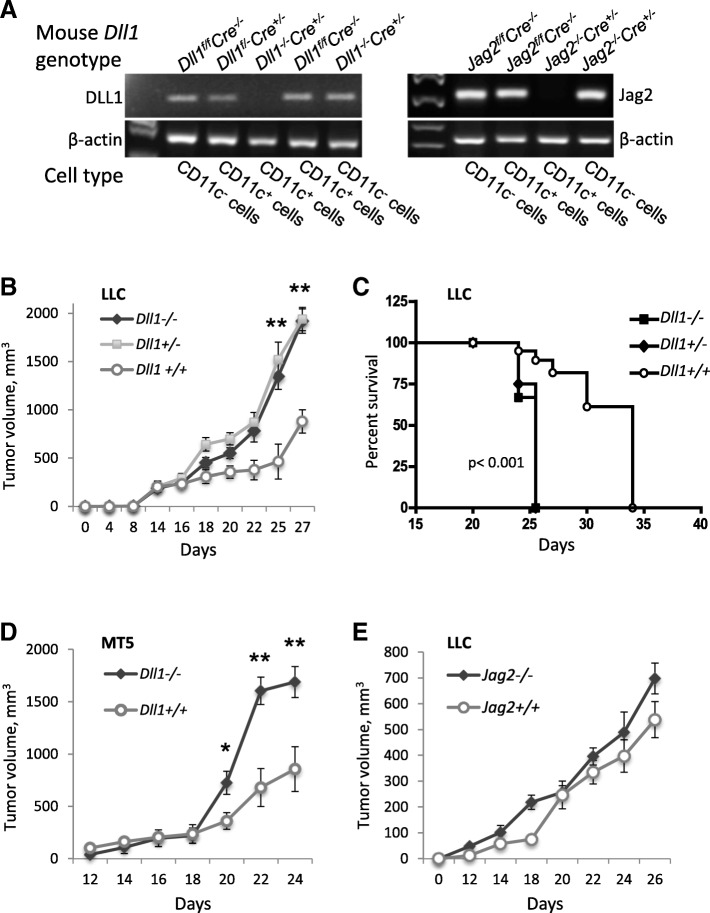
Genetic ablation of *Dll1* in CD11c^+^ cells in mice accelerates tumor growth with decreased survival. **a** Deletion of Notch ligand genes *Dll1* and *Jag2* in CD11c^+^ cells was confirmed by RT-PCR performed with RNA isolated from CD11c^+^ or CD11c^−^ cells from splenocytes of genetically modified and wild-type mice. Lewis lung carcinoma (LLC) tumor growth (**b**) and log-rank survival curves (**c**) for mice with hetero or homozygous deletion of *Dll1* in CD11c^+^ cells and wild-type littermates. **d** Pancreatic MT5 tumor growth in CD11c^+^ cell-specific *Dll1*^*−/−*^ and wild-type mice. **e** LLC tumor growth in CD11c^+^ cell-specific *Jag2*^*−/−*^ and wild-type littermates. Mean ± SEM, 8–10 mice per group; *, *p* < 0.05; **, *p* < 0.01

To evaluate the roles of Notch ligands DLL1 and Jag2 expression on DCs in the regulation of T-cell-mediated anti-tumor immunity, we generated mice with CD11c-lineage-specific deletion of their genes. Mice with hetero- or homozygous allele deletion of *Dll1* or *Jag2* appeared normal in gross morphology with respect to their wild type littermates with floxed alleles, *DLL1*^*flox/flox*^ or *Jag2*^*flox/flox*^. A representative mRNA analysis of the respective Notch ligands in flow-sorted CD11^+^ DC populations from wild type and genetically modified mice is shown (Fig. 1a). Transcripts for the tested Notch ligand mRNAs were absent in CD11c^+^ cells but present in CD11c^−^ splenic cells or whole splenocyte populations from mice with homozygous deletion of the ligands.

Genetically modified mice were inoculated subcutaneously with lung LLC or pancreatic MT5 tumor cells. Mice with hetero- or homozygous deletion of *Dll1* allele in CD11c^+^ cells exhibited remarkably accelerated LLC tumor growth and significantly decreased survival compared to their wild-type littermates (Fig. 1b, c). The effect was reproduced in the MT5 tumor model (Fig. 1d). The fact that loss of even one *Dll1* allele produced significantly accelerated disease indicates the importance of DLL1 expression on DCs for tumor rejection.

In contrast, deletion of both alleles of *Jag2* did not result in major alteration in LLC tumor growth (Fig. 1e). There was a tendency toward increased tumor volume, which was not statistically significant. These results suggest that abrogation of DLL1 but not Jag2 presentation by DC may result in an altered anti-tumor immunity that could affect tumor rejection.

### Impaired anti-tumor T-cell IFN-γ production in tumor-bearing mice lacking DLL1 in CD11c^+^ cells

**Fig. 2:**
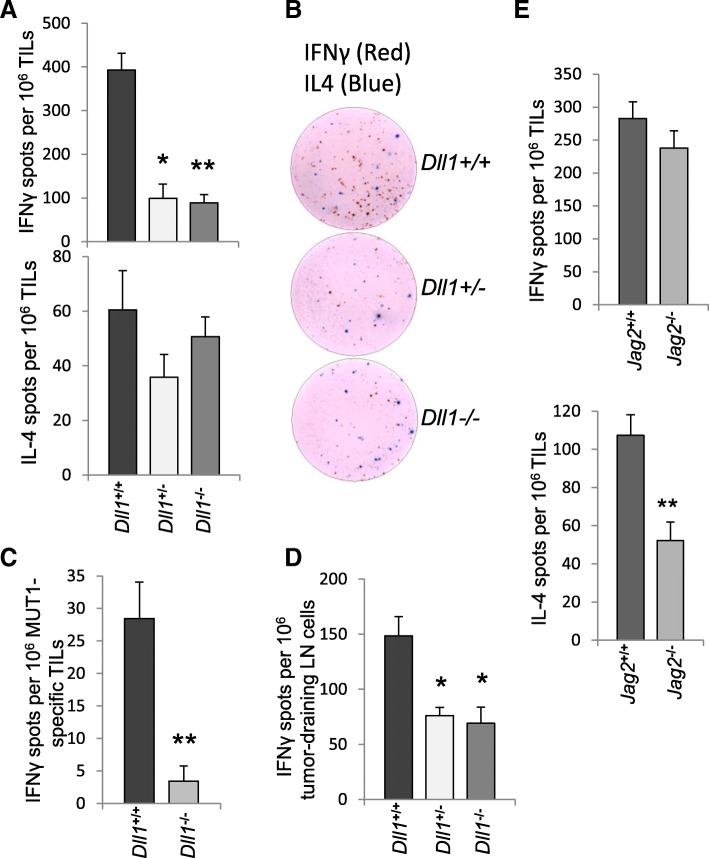
CD11c-lineage-specific ablation of Notch ligands alters cytokine production. IFN-γ and IL-4-producing cells were enumerated by ELISPOT assay among LLC tumor-infiltrating lymphocytes (TIL) from mice with CD11c lineage-specific deletion of *Dll1* and wild-type littermates following re-stimulation with anti-CD3/CD28 beads (**a, b**) or with LLC tumor antigenic peptide MUT1 (FEQNTAQP) loaded on autologous splenocytes for 48 h (**c**). **d** Evaluation of IFN-γ-producing cells in a pool of tumor-draining lymph node cells from the same mice following re-stimulation with anti-CD3/CD28 beads. **e** Evaluation of IFN-γ and IL-4-producing cells among tumor-infiltrating lymphocytes from *Jag2*^*−/−*^ or wild-type littermate mice following re-stimulation with anti-CD3/CD28 beads. Mean ± SEM, 5 mice per group; *, *p* < 0.05, **, *p* < 0.01

To test whether genetic ablation of specific Notch ligands in DCs affected cytokine secretion patterns, we evaluated IFN-γ and IL-4 production in tumor-infiltrating T-cells by ELISPOT assay following restimulation with CD3/CD28 antibody activator beads or with LLC tumor antigenic MHC class-I-restricted peptide MUT1 loaded on autologous splenocytes. We found that in mice with hetero and homozygous deletion of *Dll1* in DCs, the numbers of IFN-γ-producing MUT1-specific lymphocytes were markedly decreased in the tumor, whereas the numbers of tumor-infiltrating IL-4-producing cells were not altered (Fig. 2a-c). Similarly, a reduction of IFN-γ-secreting lymphocytes was observed in the tumor-draining lymph nodes (LN) (Fig. 2d). These observations provided the explanation for the observed differences in tumor growth rates by pointing to the crucial role of DC-expressed DLL1 for the induction of anti-tumor cytotoxic T-cell responses.

In contrast to the striking effect of *Dll1* deletion on IFN-γ production, genetic ablation of *Jag2* in DCs did not have a major effect on the number of tumor-infiltrating IFN-γ-producing cells. Abrogation of Jag2-mediated signaling, however, resulted in the decreased generation of IL-4-secreting cells (Fig. 2e) consistent with the reported role of Jag2 in Th2-type differentiation.

### Effects of CD11c^+^ cell lineage-specific deletion of *Dll1* on myeloid and lymphoid populations in tumor-bearing mice

**Fig. 3:**
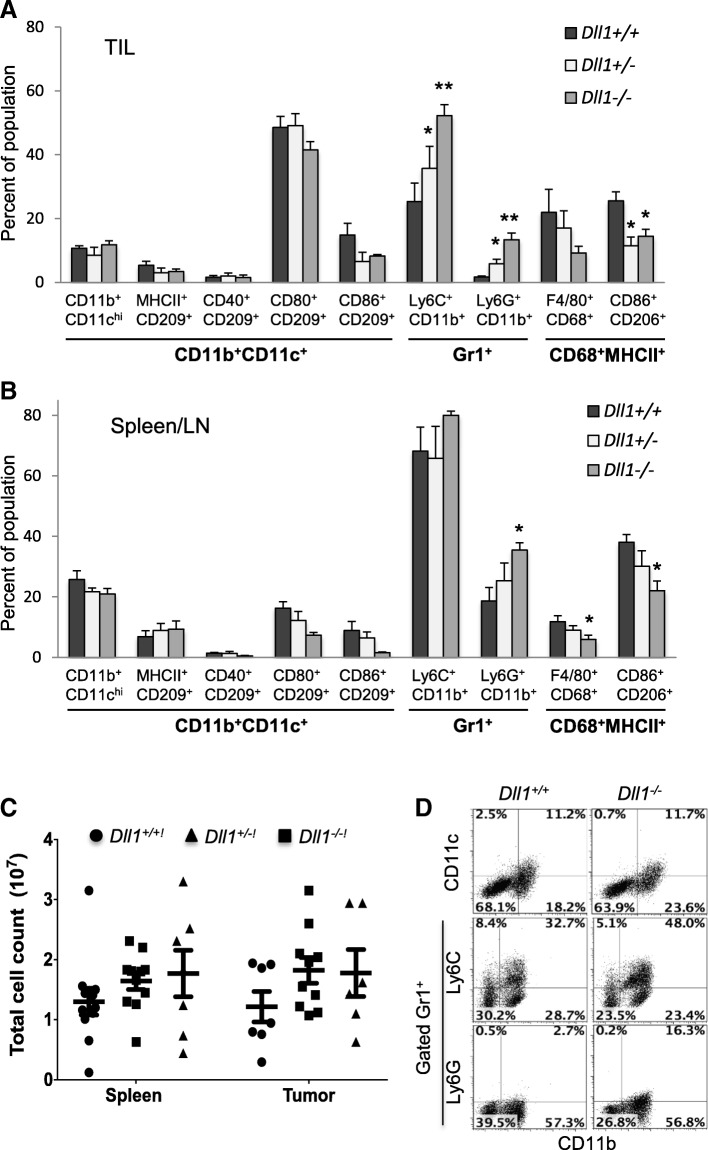
CD11c-lineage-specific *Dll1-*ablated tumor-bearing mice exhibit no change in dendritic cells but increase CD11b^+^Gr1^+^ cell proportions. Myeloid populations were evaluated by flow cytometry on day 17–18 after LLC tumor initiation in *Dll1* knockout and wild-type littermate mice. Percentage of indicated populations are shown in the tumor infiltrate (TIL) (**a**) and in a pool of splenocyte and LN cells (**b**). **c** Total cell yields in the splenocytes and tumor single cell suspensions. **d** Representative FACS plots for CD11b versus CD11c, Ly6C or Ly6G staining (**c**). Mean ± SEM, 5–7 mice per group; *, *p* < 0.05; **, *p* < 0.01

We performed extensive immunophenotyping of myeloid and lymphoid populations infiltrating the tumors and in spleens of mice with CD11c^+^ lineage-specific hetero or homozygous deletion of *Dll1* and their wild-type littermates on day 17–18 after LLC tumor establishment. The deletion of either one or two alleles of *Dll1* resulted in a moderate increase in total cell counts of tissue-resident CD11b^+^CD11c^+^ DC populations in the tumor or spleen compared to wild-type littermates, but ability of DCs to undergo maturation or infiltrate the tumor was not affected (Fig. 3a-c). Numbers of CD11b^+^CD11c^+^ DCs expressing maturation markers MHCII, CD40, CD80, CD86 and CD209 also did not change (Fig. 3a, b, d). This is consistent with the hypothesis that the observed alterations in anti-tumor T-cell responses is due to the absence of DLL1 expression on DCs. Other obvious changes in the myeloid compartment included increased numbers of CD11b^+^Gr-1^+^ cells in *Dll1-*ablated mice. Further characterization showed that both Ly6C^+^ monocytic and Ly6G^+^ granulocytic populations of CD11b^+^Gr-1^+^ cells were significantly higher in the tumors of DC-*Dll1-*ablated mice (Fig. 3a, d). In the lymphoid organs of spleen and LN also, increased numbers of CD11b^+^Gr-1^+^Ly6G^+^ granulocytic cells were observed (Fig. 3b). On the other hand, a decline was noted in the proportions of CD68^+^MHCII^+^F4/80^+^ M1 and CD68^+^MHCII^+^CD86^+^CD206^+^ M2 macrophages in the tumor-infiltrate and spleen of *Dll1-*ablated mice (Fig. 3a, b).

**Fig. 4:**
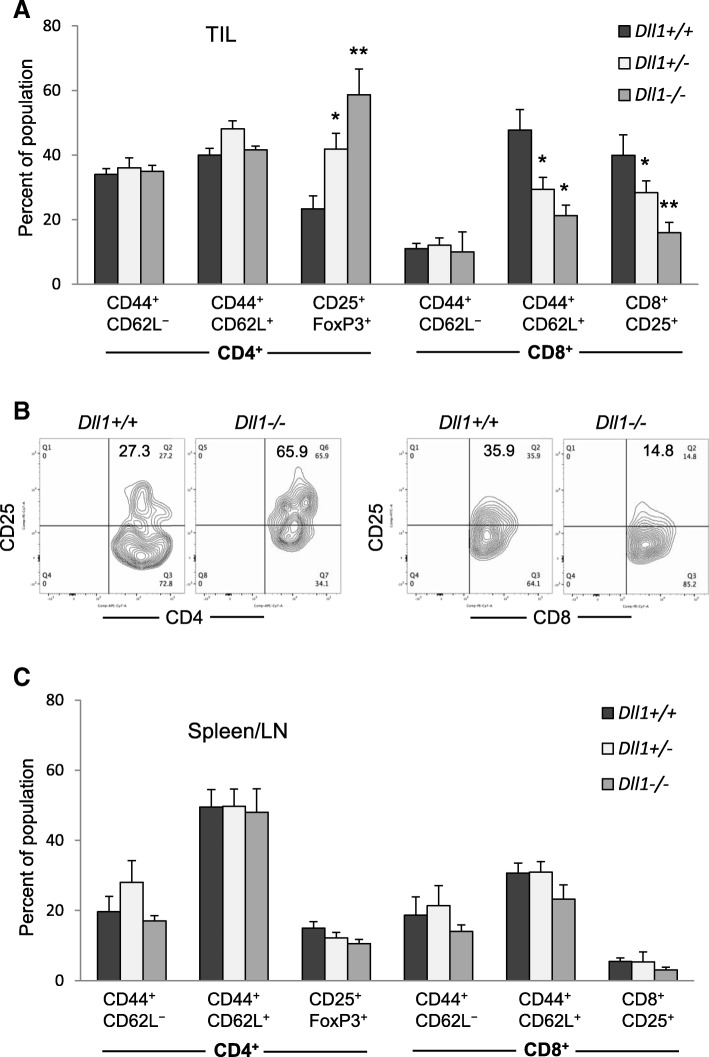
Tumor-bearing mice with CD11c-lineage-specific deletion of *Dll1* exhibit increased Treg and decreased effector T-cell subsets. Lymphoid populations were evaluated by flow cytometry on day 17–18 after LLC tumor initiation in *Dll1* knockout and wild-type littermate mice. Percentage of indicated populations are shown in CD4^+^ and CD8^+^ subsets in the tumor infiltrate (TIL) from knockout and wild-type littermates (**a**) with representative flow plots for CD4 versus CD25, and CD8 versus CD25 (**b**) and in a pool of splenocytes and LN cell cells from the same mice (**c**). Mean ± SEM, 5 mice per group, *, *p* < 0.05; **, *p* < 0.01

Among lymphoid populations, a significantly increased population of CD4^+^CD25^+^ T-cells was observed in *Dll1-*ablated mice. Most of the tumor-infiltrating CD4^+^CD25^+^ cells expressed FoxP3, and their proportion was 2-fold higher in DC-specific *Dll1*^*−/−*^ mice compared to wild-type littermates (Fig. 4a, b). Deletion of *Dll1* in DCs also had a significant effect on CD8^+^ T-cells that resulted in their decreased activation in the tumor as shown by decreased expression of CD25 and CD44. The proportions of intratumoral activated CD25^+^ and central-memory CD44^+^CD62L^+^ CD8^+^T-cells were significantly lower in knockout mice than in wild-type animals (Fig. 4a, b). These effects were, however, not prominent in splenocytes (Fig. 4c) suggesting that the observed effects of *Dll1* gene deletion may only be specific to the tumor microenvironment.

The above data show that abrogation of *DLL1*-mediated signaling favors Treg differentiation and accumulation, and suppresses effector CD8^+^T-cells in the tumor. These data indicate that genetic ablation of the DC-expressed DLL1 affects T-cell differentiation and activation programs to interfere with the generation of effective anti-tumor immune responses.

### Pharmacological interference or enhancement of DLL1-Notch signaling affects T-cell proliferation and effector responses

**Fig. 5:**
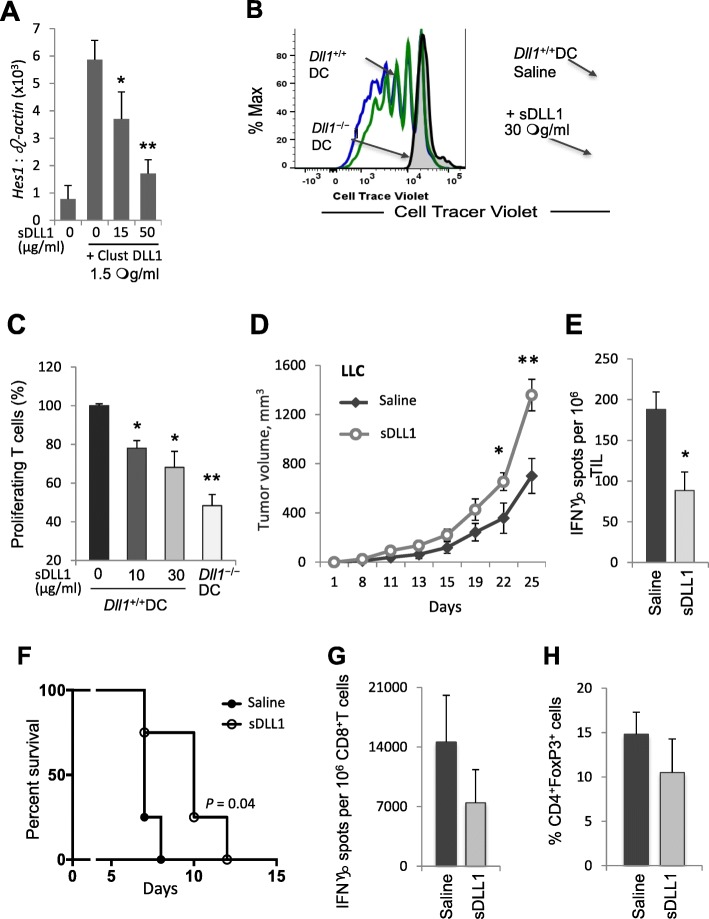
Monomeric soluble DLL1 or *Dll1*-ablated dendritic cells restrict Notch signaling and impair T-cell cytotoxic responses. (**a**) Expression of Notch downstream target *Hes1* mRNA was assessed by qRT-PCR in 3 T3 cells treated with clustered DLL1 in the presence of soluble DLL1 (sDLL1) construct at indicated concentrations for 16 h. **b**, **c** T-cell proliferation was measured after co-incubating allogeneic T-cells labeled with Cell Tracer Violet fluorescent dye with bone marrow-derived *Dll1*^*−/−*^ or wild-type DC in the presence of soluble anti-CD3 for 5 days. In some T-cell cultures with wild-type DC, soluble DLL1 construct was added at the indicated concentrations. Representative Cell Tracer Violet dye dilution profile is shown (**b**). **d** Tumor volume was measured in LLC tumor-bearing mice treated with sDLL1 construct 1 mg/kg body weight, i.p. every 2 days for 20 days. **e** IFN-γ producing tumor-infiltrating cells from these mice were enumerated by ELISPOT assay on day 18 after LLC tumor initiation. Mean ± SEM, 8 mice per group; *, p < 0.05; **, *p* < 0.05. **f, g** C57BL/6 mice were transplanted with BALB/c heart allografts on day 0 and treated with sDLL1 construct (1 mg/kg) i.p. on days − 3, − 1, 1, 3, 5 and 7. **f** Heart allografted C57BL/6 mice log-rank survival. **g** IFN-γ ELISPOT assay on recipient CD8^+^T cells isolated after heart allograft and re-stimulated with mitomycin C-treated donor spleen cells in the presence of recipient C57BL/6 splenocytes. **h** Percentage of FoxP3^+^ cells among CD4^+^ splenocytes after heart allograft. Mean ± SEM, 4–8 mice per group; *, p < 0.05

Activation of Notch receptor proteolytic cleavage and signaling requires a context-dependent multivalent interaction between Notch receptors and ligands, whereas soluble monovalent forms of ligands are known to inhibit Notch signaling [24, 29]. We engineered a monovalent soluble DLL1 construct (sDLL1) comprising one DSL and two N-terminal EGF repeat domains, and compared its effects with multivalent clustered DLL1, a complex formed by DLL1-IgG Fc fusion protein, biotinylated anti-Fc antibodies and avidin that selectively triggers DLL1-Notch signaling [24]. The monomeric sDLL1 construct inhibited Notch signaling triggered by multivalent clustered DLL1 as manifested by the dose-dependent decrease in expression of the Notch downstream target *Hes1* mRNA in treated murine 3 T3 fibroblast cells (Fig. 5a). Thus, sDLL1 acts as a competitive inhibitor of multivalent  DLL1-triggered signaling.

To further confirm that DLL1-mediated Notch signaling is required for efficient T lymphocyte function, we assessed the effects of genetic versus pharmacological interference with DLL1 signaling on the ability of DCs to stimulate T-cell proliferation and function. DCs were generated from wild-type or DC-specific *Dll1*^*−/−*^ mouse bone marrow cells, as described [30], and co-cultured with allogeneic T-cells labeled with cell tracer fluorescent dye in the presence of soluble CD3 antibody. In T-cell co-cultures with wild-type DCs, sDLL1 protein was added to block DLL1-mediated signaling. DCs generated from *Dll1*^*−/−*^ mouse showed impaired ability to stimulate T-cell proliferation in contrast to wild-type DCs. The presence of sDLL1 in T-cell stimulation cultures also resulted in decreased T-cell proliferation as assessed by the cell tracer dye dilution profile (Fig. 5b, c). Moreover, LLC tumor-bearing wild-type mice treated with sDLL1 significantly increased tumor growth (Fig. 5d), similar to accelerated tumor growth seen in DC-specific *Dll1*^*−/−*^ mice (Fig. 1b). The inhibition with sDLL1 also resulted in decreased IFN-γ-producing tumor-infiltrating T-cells (Fig. 5e). Thus, pharmacological or genetic disruption of DLL1-Notch interaction decreased T-cell proliferation, IFN-γ production and anti-tumor T-cell function, confirming the critical requirement for DLL1-Notch signaling for DC-supported T-cell function.

We further tested the effect of interference with DLL1-mediated Notch activation on the induction of T-cell cytotoxic responses using the sDLL1 construct in the non-tumor context of a cardiac allograft rejection. C57BL/6 mice were transplanted with MHC-mismatched heterotopic BALB/c heart allografts and treated with sDLL1 construct (1 mg/kg body weight, i.p.) or vehicle control every 2 days. The sDLL1 treatment of C57BL/6 recipient mice significantly prolonged host allograft survival when compared to mice treated with vehicle control (Fig. 5f). This prolonged survival was associated with a decreased production of IFN-γ by donor-reactive recipient C57BL/6 CD8^+^ T-cells following restimulation with mitomycin-C-treated allogenic donor BALB/c splenocytes in the presence of recipient antigen-presenting cells (Fig. 5g). There was no major shift toward IL-4 or IL-17 production (82 ± 7 vs. 84 ± 8 IL-4 spots and 16 ± 5 vs. 18 ± 2 IL-17 spots per 10^6^ spleen cells for control and sDLL1-treated groups, respectively), or in the proportion of CD4^+^FoxP3^+^ T-cells (Fig. 5h). These data demonstrate that interference with the DLL1-mediated Notch activation specifically suppresses induction of anti-tumor or alloreactive T-cell responses.

**Fig. 6:**
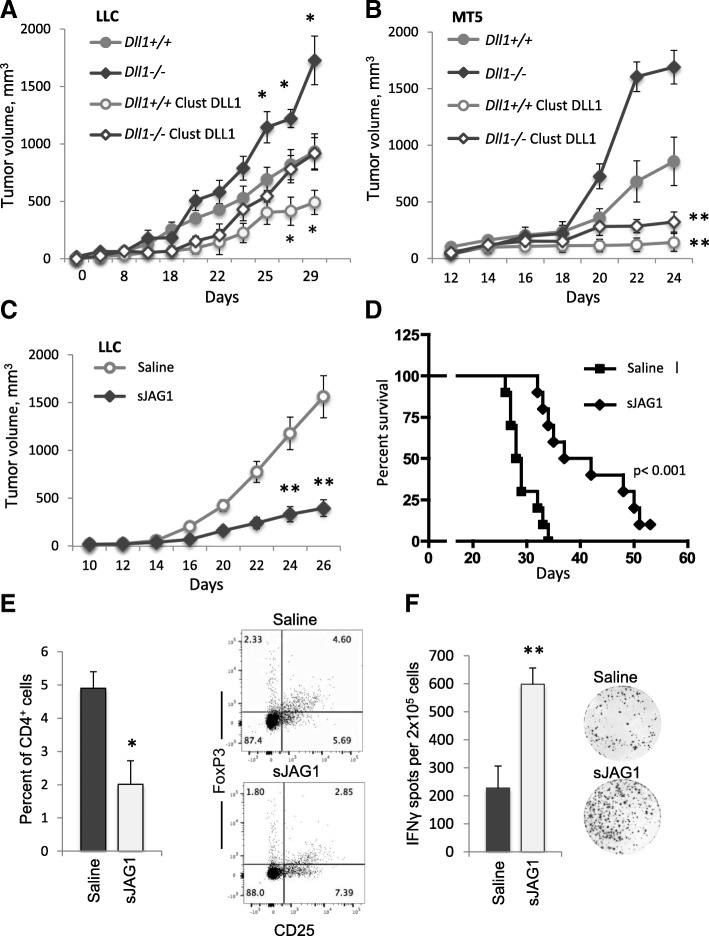
Pharmacological manipulation of DLL1 or Jag1-mediated signaling reduces tumor growth and improves anti-tumor immunity. **a**, b Enhancement of DLL1 signaling using multivalent clustered DLL1 overcomes the critical dendritic cell DLL1 deficiency and restricts tumor growth. Growth of LLC tumor (**a**) and MT5 pancreatic tumor (**b**) in wild-type and DC-specific *Dll1*^*−/−*^ mice. Mice were treated with 0.2 mg/kg body weight of multivalent clustered DLL1-Fc fusion protein every 2 days for 20 days. Mean ± SEM, 8 mice per group; *, p < 0.05; **, *p* < 0.01. **c**, **d** Treatement with soluble fragment of extracellular domain of JAG1 (sJAG1) significantly reduces tumor growth and improves survival of tumor-bearing mice. LLC tumor growth (**c**) and log-rank survival curves (**d**) in mice treated with monovalent soluble JAG1 construct, 1 mg/kg body weight, i.p. every 2 days for 20 days. Percentage of FoxP3^+^ cells among CD4^+^ cells (**e**) and IFN-g ELISPOT (**f**) for splenocytes in mice treated with soluble JAG1 on day 18 after LLC tumor initiation. Mean ± SEM, 8–10 mice per group; *, *p* < 0.05; **, *p* < 0.01

In search for potential therapeutics to correct insufficiency or deficiency in DC-expressed DLL1-mediated Notch activation pharmacologically, we tested the efficacy of multivalent clustered DLL1 in the setting of DLL1 deficiency in tumor-bearing hosts. The wild-type or DC lineage-specific *Dll1*^*−/−*^ mice were established with lung LLC or pancreatic MT5 tumors and treated with clustered DLL1 every two days for twenty days. Clustered DLL1 treatments significantly reduced tumor growth and prolonged survival in both wild-type and DC-specific *Dll1*^*−/−*^ animals (Fig. 6a, b).

These results with monomeric or clustered DLL1 constructs demonstrate that DLL1-based therapeutics has the potential to attenuate or activate Notch signaling in various disease conditions. Soluble DLL1 can prevent allograft rejection, whereas clustered DLL1 can substitute in large part for inadequate presentation of DLL1 by DCs needed for proper T-cell stimulation, and elicit anti-tumor responses to reject tumors.

### Monomeric soluble JAG1 construct decreases Treg frequency, reduces PD-1 expression on CD8^+^Tem cells and improves anti-tumor immunity

Our results showing differential cytokine patterns in mice with DC-specific deletion of *Dll1* and *Jag2* ligands suggested that Notch ligands had differential effects on the induction of immune responses. We constructed a monovalent soluble JAG1 (sJAG1) construct comprising total five N-terminal domains (MNNL, DSL and 3 EGF repeats) of mouse JAG1, and evaluated the significance of JAG1-mediated Notch signaling in anti-tumor responses. LLC tumor-bearing mice were treated with sJAG1 at a dose of 1 mg/kg body weight or vehicle control, i.p. every 2 days. The treatment with sJAG1 resulted in significant reduction of tumor growth and improved survival of animals (Fig. 6c, d). Moreover, these effects were associated with the decreased numbers of CD4^+^FoxP3^+^ Treg cells. We also noted significantly reduced accumulation of tumor-infiltrating CD11b^+^Gr1^+^ cells (40.1 ± 8.3% vs. 11.0 ± 3.8% among CD45^+^ cells for control and sJAG1-treated groups, respectively), and increased IFN-γ production by lymphoid cells (Fig. 6f). These data suggest an important role of JAG1-mediated Notch signaling in the induction of anti-tumor T-cell responses.

**Fig. 7:**
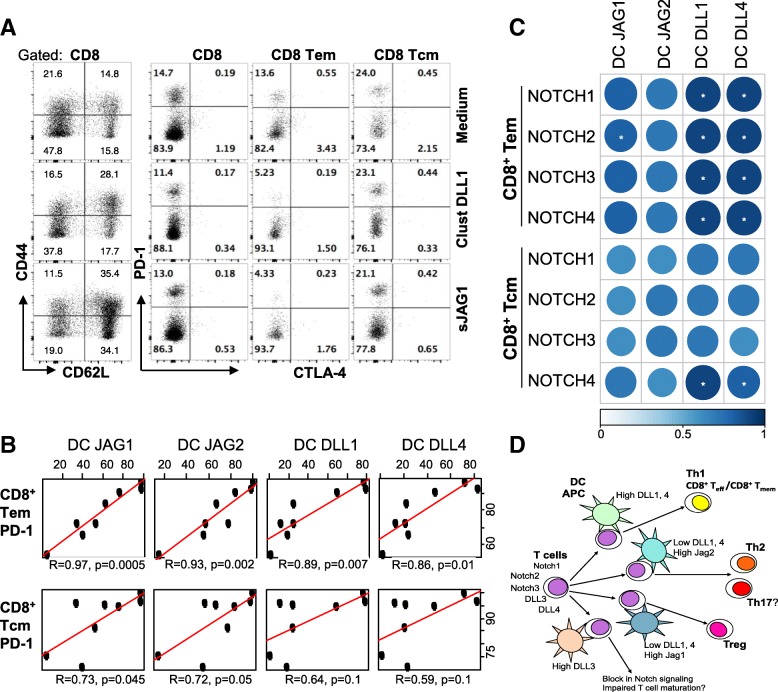
Dendritic cell Jagged expression correlates with PD-1 expression on T-effector-memory cells. **a** Purified T cells were stimulated in vitro in a T:DC (3:1) stimulation co-culture with allogeneic bone marrow-derived dendritic cells in the presence of CD3/CD28 beads (1 μg/mL) for four days with or without treatment with clustered DLL1 (1.5 μg/mL) or monovalent soluble JAG1 (20 μg/mL) constructs. Expression of CD62L, CD44, CTLA-4 and PD-1 was assessed on gated populations as indicated by flow cytometry. Dot plots from a representative experiment out of two independent experiments with duplicates are shown. **b**-**c** Lung tumor single cell suspensions from 10 patients were evaluated for the expression of NOTCH ligands on tissue-resident CD11b^+^CD11c^high^ dendritic cells and PD-1 and NOTCH receptors in populations of T cells by flow cytometry. NOTCH ligands in CD11b^+^CD11c^high^ cells were compared to PD-1 positivity of Tem and Tcm cells (**b**) or to NOTCH receptor positive T cell subsets by Pearson’s correlation (**c.**) All *p*-values were corrected using the Benjamani- Hochberg procedure; *n* = 8; * p < 0.05. Color code indicates the strength of correlation. **d** Scheme summarizing available data on the regulation of T cell responses by Notch ligands

We also assessed whether the engineered Notch ligand DLL1 and JAG1 constructs modulate the differentiation of memory T-cells in vitro in a T:DC stimulation co-culture. Results show that inhibition with soluble JAG1 or stimulation with clustered DLL1 constructs increased the frequency of CD8^+^T-central memory cells concomitant with a decrease in the frequency of CD8^+^T-effector memory cells (Fig. 7a and Additional file 1: Figure S1). The decrease in the frequency of CD8^+^Tem cells was not due to exhaustion as both constructs significantly decreased the expression of checkpoint molecule PD-1 (about 3-fold by sJAG1 and 2.5-fold by clustered DLL1) in CD8^+^Tem cells, but not in CD8^+^Tcm, in a dose-dependent manner (Fig. 7a and Additional file 2: Figure S2). The expression of CTLA-4 was negligible and unchanged following treatments with the constructs.

### Dendritic cell-expressed NOTCH ligands correlate with PD-1 or NOTCH receptor expression in CD8^+^ T-effector memory population in human lung tumor-infiltrates

We sought to determine if there is a relationship between the expression of NOTCH ligands on antigen presenting cells and T-cell phenotype in human lung cancers. We profiled the expression of NOTCH ligands, NOTCH receptors and PD-1 on various subsets of myeloid and lymphoid cells in tumor-infiltrating immune cells in primary lung cancers. The analysis revealed a highly significant correlation between the proportion of JAG1 or JAG2-expressing tissue-resident CD11b^+^CD11c^high^ DCs and the numbers of PD-1-expressing T-effector-memory (Tem) CD8^+^CCR7^−^CD45RA^−^ and T-terminal-effector (Temra) CD8^+^CCR7^−^CD45RA^+^ cells, with JAG1 demonstrating the strongest correlation (*p* = 0.0005) (Fig. 7b and Additional file 3: Figure S3). Correlations between DC-expressed DLL1 (*p* = 0.007) or DLL4 (*p* = 0.01) and PD-1 on Tem subsets were also observed; however, significance of these correlations was substantially less than that for JAG1 (Fig. 7b). Correlations between CD11b^+^CD11c^high^ cells expressing JAG ligands and PD-1-expressing CD8^+^CCR7^+^CD45RA^−^ Tcm were marginal, with no significant correlation between PD-1 in Tcm and Delta-like ligands (Fig. 7b).

In contrast to PD-1, numbers of Tem cells expressing NOTCH receptors highly significantly correlated with the proportion of DCs expressing DLL1 or DLL4 (Fig. 7c). While statistically significant correlation of Tem-expressed NOTCH2 and NOTCH3 was observed with DC-expressed JAG1 and JAG2, it was less pronounced. No statistically significant correlations were identified for Tcm cells, except for NOTCH4 receptor with DC-expressed JAG1, DLL1 and DLL4 (Fig. 7c). There was no correlation between NOTCH ligand expression on DCs and expression of PD-1 or NOTCH receptors in the populations of CD8^+^CCR7^+^CD45RA^+^ naïve CD8 T cells or naïve, effector or memory CD4 T-cells (Additional file 3: Figure S3). The above results imply that the interactions between select DC-expressed Notch ligands and Notch receptors in T-cells present a key point of regulation for T-cell-mediated immunity by modulation of T-cell differentiation and functionality in human lung tumors.

## Discussion

Interaction of DCs with T lymphocytes is critical to determining the type and strength of the induced immune response. Adequate presentation of antigens along with other essential signals and cytokines provided by DCs are necessary for the differentiation of effector T-cells and to elicit a strong anti-tumor immunity. It is known that distinct inflammatory responses up-regulate expression of either Delta-like or Jagged ligands in DCs to guide activated CD4^+^ T-cells toward a specific type of T helper commitment [12, 18]. The current study demonstrates that in addition to the known T-cell differentiation signals, the interaction between selective Notch ligands presented by DCs and Notch receptors on T-cells provide critical differentiation signals, which function to polarize lymphocytes towards T effector and memory cells. Our data show that presentation of DLL1 by DCs is indispensable for the induction of anti-tumor T-cell responses.

Notch signaling is highly responsive to variation in Notch ligand expression in hematopoietic organs [31–33]. Previous studies showed that altered expression of Notch ligands could underlie immunosuppression in cancer, and in particular, expression of Delta-like ligands DLL1 and DLL4 was significantly down-regulated in tumor-bearing hosts [20, 24, 25]. DC lineage-specific genetic ablation or systemic blockade of DLL1-Notch interaction, as shown in this study, resulted in accelerated tumor growth in the tested lung and pancreatic tumor models, likely due to insufficient DLL1 signaling and consequent impairment of anti-tumor immune responses. Deficiency in DLL1 expression in DCs resulted in a significant reduction of CD8^+^ T-cell activation, tumor antigen-specific CTL and differentiation of central memory CD8^+^CD44^+^CD62L^+^ T-cell populations. The DLL1 deficiency was also associated with an accumulation of monocytic and granulocytic CD11b^+^Gr1^+^ cells and increased differentiation of Treg cells. The results imply that the adequate expression of DLL1 in DCs is a prerequisite for eliciting effector T-cells and efficient anti-tumor responses.

Notch can orchestrate multiple T-cell lineage programs and concurrently regulate Th1, Th2 and Th17 cell differentiation. In this function, Notch activity is thought to be unbiased or unaffected by the cytokine environment [34]. Our study reveals that cell-lineage specific ligand-receptor interactions determine the T-cell lineage commitment and effector outcomes. Our findings in mice with DC-specific ablation of Notch ligands and therapeutic modulation in tumor and allograft rejection settings by engineered ligand constructs support the earlier gain-of-function observations [17, 18], and strongly suggest the instructive nature of the interactions between DC-expressed Notch ligands and T-cell Notch receptors in regulating T lymphocyte commitment and effector responses.

Notch1 and Notch2 have been identified as key Notch receptors for eliciting T-cell effector function, including anti-tumor responses. It was recently shown that Notch1 activation occurred in peripheral CD4^+^ T-cells in a ligand-independent manner through chemical alterations in the endosome within a few hours post–TCR stimulation and was required for optimal T-cell activation [35]. Another study revealed the involvement of Notch signaling in the regulation of T-cell metabolic reprogramming and proposed that activation of Notch1 on Th cells by DC-expressed DLL4 was essential for fine tuning the sensitivity, magnitude and quality of the initial CD4^+^ T-cell responses upon antigen encounter [21]. Given the confirmed functions of Notch1, Notch2 and DLL4 in DC-T-cell interactions along with our data on the involvement of DLL1 in Th cell polarization and CD8^+^ T-cell differentiation into effector and memory cells, it is reasonable to hypothesize that the roles of different Notch receptors and ligands are distinct during the multistep process of T-cell lineage commitment and differentiation. The initial interaction between DC-expressed DLL4 and T-cell Notch1 would support T-cell activation and metabolic reprogramming, enhance expression of Notch2 and potentially modulate the expression of other Notch ligands. Engagement of Notch2 and DLL1 then would drive effector T-cell differentiation and CTL responses. Altogether, the results point to a functional axis of DLL4/DLL1 and Notch1/Notch2 as an essential element in DC-T-cell interactions needed for the induction of effector T-cell differentiation and eliciting T-cell-mediated anti-tumor immunity.

Among Jagged family of Notch ligands, Jag2 was previously implicated in the induction of Th2 type responses [16, 19, 33]. In our study, *Jag2* deletion in DCs did not result in any major changes in anti-tumor T-cell responses, such as IFN-γ production but had a negative effect on the number of IL-4 producing cells, consistent with the role of Jag2 in supporting Th2 differentiation. In contrast, pharmacological treatment with monomeric soluble JAG1 resulted in remarkable inhibition of tumor growth that was associated with the down-regulation of Treg cell differentiation, significantly decreased tumor infiltration with CD11c^+^Gr1^+^ cells, and enhanced IFN-γ production. Together with recently published data implicating Jag1 in regulating the suppressive function of myeloid-derived suppressor cells (MDSC) [36], these data identify Jag1 signaling as a prominent factor in immunosuppression mediated by both regulatory T-cells and MDSC.

Analysis of human tumor-infiltrating immune cells confirmed the potential link between expression of Notch ligands by tissue-resident CD11b^+^CD11c^high^ DCs and functional state of T-cells defined by their expression of PD-1 and Notch receptors. PD-1 expression is regulated by multiple intercellular interactions, including Notch-mediated transcriptional control of the *Pdcd1* gene encoding PD-1 in CD8^+^ T cells [37]. Recently, stem cell memory (scm)-like T-cells were generated from activated murine and human CD4^+^ and CD8^+^ T-cells by coculturing with stromal cells presenting DLL1 ligand. Further, Notch-mediated conversion of activated cells into Tscm was associated with the loss of PD-1 and CTLA-4 molecules [38]. We also show enhancement of CD8^+^ Tcm populations with a decrease of PD-1 expression by clustered DLL1-triggered signaling or inhibition of JAG1-mediated signaling. Our data have identified a novel link between PD-1 expression in effector-memory T-cells and expression of Jagged ligands by DCs. This Jagged-PD-1 axis is consistent with the inhibition of anti-tumor T-cell activity and prevents the induction of lasting T-cell-memory responses. Data also stress high significance of Jag1 as a therapeutic target and indicate that its blockade would be beneficial through multiple mechanisms including decreased expression of PD-1 in T-cells. Significance of the regulation of Notch receptors in Tem cells by DC-expressed Notch ligands is yet to be elucidated.

Available data on the roles of different Notch ligands in regulation of T-cell differentiation are summarized in Fig. 7c. With both Notch1 and 2 receptors involved, higher DLL1 and DLL4 expression by DCs and other antigen-presenting cells favors Th1 type and CD8^+^ CTL responses [14, 24, 25]. Higher expression of Jag2 is linked to predominant Th2 and likely Th17 type responses, whereas high expression of Jag1 and decreased expression of Delta-like ligands supports regulatory T-cell commitment [8, 15, 16, 19, 33, 39, 40].

The essential role of Notch ligands in the immune regulation raises an important question about the factors that modulate their expression in DCs. A number of factors affecting expression of Notch ligands have been identified in various cellular and tissue systems [41]. Some of them, including VEGF, FGF and PGE2, have been implicated in the generation of dysfunctional or immunosuppressive DCs. It is conceivable that part of these immunosuppressive effects could be mediated via alteration of Notch ligand presentation and shift from ligands critical for Th1 or CTL differentiation toward ligands committed to other T-cell lineages including Treg and Th2.

Identification of pharmacological approaches to modulate ligand-specific Notch signaling for the therapeutic induction of immune responses could provide a powerful tool for directing polarization of T lymphocytes and dissecting the T-cell differentiation requirements. We tested approaches to modulate Th1 type, CTL and Treg responses using multivalent activating or monovalent inhibiting DLL1 and JAG1 constructs in lung and pancreatic tumor and cardiac allograft models. Therapeutic activation of Notch signaling by clustered DLL1 in large part restored deficient presentation of DLL1 by DCs. Conversely, interference with ligand specific signaling by monovalent soluble JAG1 or soluble DLL1 efficiently improved anti-tumor immunity or blocked anti-tumor and allogeneic T-cell responses, respectively. The experiments with engineered mono- and multivalent Notch ligands demonstrate the potential of Notch ligand-based constructs in regulation of specific types of immune responses and open a venue for exploration of a novel class of therapeutics for modulating immunity.

## Conclusions

We studied the effects of CD11c lineage-specific deletion, inhibition or activation of Notch ligands on T-cell function. Distinct immunoregulatory roles of Notch ligands were identified, with dendritic cell-expressed DLL1 and JAG1 having opposing effect on CTL responses. Results suggest that engineered Notch ligand constructs could be a novel class of immunomodulatory drugs. However, no direct data are yet available to show the efficacy of such Notch ligand constructs to human cancers. Nevertheless, the consensus is building that regulation of Notch in cancer immunity is a very attractive approach [42] that will further enhance therapeutic success in the ongoing revolution in cancer immunotherapy [43, 44]. Thus, strategies incorporating selectively engineered Notch ligands could open a new approach of therapeutics for modulating immunity in various immunosuppressive conditions including cancer.

## Methods

### Cell lines

Murine Lewis lung carcinoma (LLC) and 3 T3 cell lines were obtained from the American Type Culture Collection (Manassas, VA). Murine MT5 pancreatic cells were a kind gift from Dr. Tuveson (Cold Spring Harbor Laboratory, Cold Spring Harbor, NY). Low-passage (less than 10) cultures were used for the experiments. All active cell cultures were checked for mycoplasma routinely using a commercial PCR test. Purity and identity of cells were also confirmed by flow cytometry (FACS) analysis with antibodies to appropriate markers.

### Mice and generation of DC lineage specific Notch ligand knockout mice

Male and female C57BL/6 and BALB/c mice (7 to 8-week-old) used at equal numbers were purchased from The Jackson Laboratory (Bar Harbor, MN).

C57BL/6 mice with floxed alleles for the *Dll1* gene were received from Dr. J. Lewis (Cancer Research UK, London, UK); *Jag2* gene-targeted floxed mice were kindly provided by Dr. T. Gridley (Maine Medical Center, Scarborough, MN). Generation of *Dll1*^*flox/flox*^ and *Jag2*^*flox/flox*^ conditional knockout mice and genotyping of floxed and deleted alleles have been described previously [45–47]. B6.Cg-Tg(Itgax-cre)1-1Reiz/J mice expressing Cre recombinase under the CD11c (integrin-αX; CD11c-Cre) promoter were purchased from The Jackson Laboratory. The animals were housed in pathogen-free units.

We generated mice bearing deletion of *Dll1* or *Jag2* in CD11c^+^ cells by mating syngeneic B6.Cg-Tg(Itgax-cre)1-1Reiz/J mice expressing Cre-recombinase under CD11c promoter and *DLL1*^*flox/flox*^ or *Jag2*^*flox/flox*^ mice and then by crossing their progeny. In the resultant mice, CD11c^+^ cells with hetero- or homozygous allele deletion had genotype *Dll1*^*flox/−*^*Cre*^*+/−*^, *Jag2*^*flox/−*^*Cre*^*+/−*^, *Dll1*^*−/−*^*Cre*^*+/−*^ or *Jag2*^*−/−*^*Cre*^*+/−*^, respectively. Their littermates with “floxed” alleles but without Cre recombinase transgene served as respective controls in all animal experiments. The allele deletion was confirmed by genotyping and by the assessment of Notch ligand mRNA expression in flow-sorted CD11c^+^DC populations from the spleen by PCR and RT-PCR using genomic DNA and RNA samples, respectively, with sets of primers specific for floxed and deleted alleles and for ligand mRNA described previously [24, 45–47] (Fig. 1a).

### Expression levels of Notch ligands

RT-PCR was utilized to confirm deletion of Notch ligand genes in CD11c^+^ cells. CD11c^+^ cells were isolated from splenocytes by flow sorting, as described below. RNA was extracted with RNeasy Mini kit and possible genomic DNA contamination was removed by on-column DNase digestion using the RNase-free DNase kit (Qiagen; Valencia, CA). cDNA was synthesized using SuperScript III Reverse Transcriptase kit (Invitrogen, Grand Island, NY) and used in PCR reactions with gene-specific primers, described previously [24]. Amplification of endogenous β-actin was used as an internal control.

### Pharmacological inhibition and activation of DLL1 and JAG1 signaling

We engineered a DLL1 construct where part of the soluble extracellular domain of mouse DLL1 protein comprising DSL, EGF1 and EGF2 domains with TEV and 6-His sequences was expressed in *E. coli* and isolated using Ni-column (Bio-Rad, Hercules, CA). The preparation was 90% pure as assessed by polyacrylamide gel electrophoresis with Coomassie R-250 staining. Mouse JAG1 construct comprising MNNL, and DSL followed by three EGF domains of JAG1 was produced in *E. coli* by MyBiosource, Inc. (San Diego, CA). The inhibitory activity of these reagents was confirmed by their ability to decrease Hes1 expression in response to Notch stimulation with the respective ligand in cell culture assay. These constructs are referred to in the text and figures as soluble DLL1 (sDLL1) and soluble JAG1 (sJAG1), respectively.

### Tumor model experiments and treatments

To induce tumors, mice were inoculated subcutaneously (s.c.) in the flank with 0.25 × 10^6^ LLC or 10^6^ MT5 cells as described previously [25, 30, 48]. Tumor volume was measured with calipers. For survival experiments, mice were observed until they reached exclusion criteria as determined by the IACUC protocol. To evaluate immunological correlates, mice were euthanized on days 17–18 and 14–15 for LLC and MT5 models, respectively.

To inhibit ligand-specific Notch signaling, tumor-bearing mice received sDLL1 or sJAG1 at a dose of 1 mg/kg body weight (25 μg per injection) of the protein in 100 μl of vehicle (Saline, 5% glycerol) intraperitoneally (i.p.) every other day. The control groups received 100 μl of saline vehicle instead of the ligand. Multivalent form of DLL1 (clustered DLL1) was utilized to stimulate DLL1-mediated Notch activation in vivo at a dose of 0.2 mg/kg body weight (5 μg per injection) of DLL1-Fc fusion protein i.p. every other day, as described previously [24, 25]. All treatments started at day 3–4 after tumor injection.

### Tumor cell suspension, lymphocyte enrichment and T-cell functional analyses

Single cell suspensions of tumors from mice were prepared using Miltenyi Biotec (Auburn, CA) Tissue dissociation kit and Gentle MACS instrument according to the manufacturer’s recommendations. Lymphocytes were than enriched by Lympholyte M (Cedarlane, Burlington, Canada) gradient centrifugation and used to quantify the cytokine producing cells. LLC cells express a defined antigenic peptide MUT1 (spontaneously mutated connexin 37), FEQNTAQP allowing evaluation of antigen-specific CD8^+^T-cell responses [49]. Briefly, 5 × 10^5^ cells per well from the tumor cell suspensions or 2 × 10^5^ cells per well from tumor-draining LN were restimulated with 10 μM of MUT1 or irrelevant control peptide loaded on autologous mitomycin-C treated splenic cells for 48 h and IFN-γ or IL-4-producing cells were evaluated by dual ELISPOT assay (CTL, Shaker Heights, OH) according to the manufacturer’s protocol. Peptides were synthesized by the American Peptide Company, Inc. (Sunnyvale, CA). Alternatively, gradient centrifugation-enriched cells (1.5 × 10^5^ cells per well) were stimulated with Dynabeads Mouse T-Activator CD3 and CD28 antibodies coupled to beads (Life Technologies, Carlsbad, CA), as recommended by the manufacturer, and IFN-γ or IL-4-producing cells were enumerated by ELISPOT assay. Parts of tumor single cell suspension, splenocytes and LN cell population from the same mice were used for immunophenotyping of cells by FACS (see below).

The effect of Notch ligand gene knockout on T-cell stimulatory activity of DCs was evaluated in allogeneic mixed lymphocyte reaction (MLR). DCs were generated from bone marrow of wild type or knockout animals in the presence of GM-CSF and IL-4, as described earlier [30]. T-cells from allogeneic mice isolated by negative selection using T-cell isolation columns (R&D Systems, Minneapolis, MN) were labeled with Cell Tracer Violet dye (ThermoFisher Sci., Grand Island, NY) and incubated for 5 days with bone-marrow derived DC in the presence of a soluble anti-CD3. Dye dilution in proliferating T-cells was measured by flow cytometry.

### Cardiac allograft rejection model

Wild type C57BL/6 mice were transplanted with MHC-mismatched heterotopic BALB/c heart allografts as previously described [50]. Mice with transplants were treated with sDLL1 construct (1 mg/kg body weight, i.p.) or vehicle control (Saline, 5% glycerol) on days − 3, − 1, 1, 3, 5 and 7 relative to the day of transplantation. Heart allograft survival was evaluated daily by palpation, and rejection was confirmed by laparotomy. IFN-γ ELISPOT assay was performed on recipient splenic CD8^+^ T-cells isolated at the time of graft rejection and restimulated with mitomycin C treated donor BALB/c spleen cells in the presence of self-antigen presenting cells.

### Patient samples and analysis of PD-1 and NOTCH proteins on tumor-infiltrating immune cells

Ten freshly resected de-identified lung cancer samples were obtained under an informed consent from an unselected patient population in terms of tumor types and stages, age, sex and ethnicity. Tumor tissue single cell suspensions were prepared using the Tissue dissociation kit and Gentle MACS instrument from Miltenyi Biotec according to the manufacturer’s recommendations. Cells were live frozen until the analysis. Analysis of tumor- infiltrating immune cells was performed by flow cytometry using lineage-specific antibodies and antibodies to PD-1, NOTCH receptors and ligands. After gating for T-cell and myeloid subsets, cell populations were further gated by PD-1, Notch ligand, or Notch receptor positivity. Populations of PD-1-positive, Notch ligand-positive, or Notch receptor-positive cells were compared using Pearson’s correlation in R using the packages “Hmisc” and “corrplot”.

### Flow cytometry and cell sorting

Fluorochrome-labeled cell surface or intracellular protein specific antibodies were obtained from BD Biosciences, Pharmingen and eBioscience, Inc. (San Diego, CA). For staining of cell-surface markers, cells were incubated with the antibodies for 20 min on ice. For intracellular FoxP3 staining, cells were first stained for lineage-specific markers, then permeabilized for 20 min with BD fixation/permeabilization kit and incubated with fluorochrome-labeled FoxP3-specific antibody. Matched fluorochrome-conjugated isotype IgG controls were used as staining controls. Flow sorting of CD11c^+^ cells from splenocytes of wild type mice or animals with Notch gene deletion was performed using Aria IIu cell sorter (BD Immunocytometry). Nonviable cells were excluded using 7-amino actinomycin D staining. Antigen negativity was defined as having the same fluorescent intensity as the isotype control. FACS data were acquired using a FACS LSR II (BD Immunocytometry) or a Guava EasyCyte HT (Millipore) instrument and analyzed with FlowJo software (Tree Star, Ashland, OR).

### Statistical analysis

Data were analyzed using the GraphPad Prism 4.0 software (GraphPad Software Inc., San Diego, CA) and presented as mean ± SEM. Comparisons between treatment and control groups were performed using one-way ANOVA followed by Dunnett’s post-tests. Comparisons between two groups were performed using two-tailed unpaired *t* tests. Survival curves were compared using Mantel-Haenszel log rank test. The *p*-values for multiple comparisons in human sample analyses were adjusted using the Benjamani-Hochberg procedure. Differences were considered statistically significant when *p*-values < 0.05.

## Additional files

Additional file 1: **Figure S1.** Clustered DLL1 and soluble JAG1 constructs modulate the differentiation of memory T-cells in vitro. Purified T cells were stimulated in vitro in a T:DC (3:1) stimulation co-culture with allogeneic bone marrow-derived dendritic cells in the presence of CD3/CD28 beads (1 μg/mL) for four days with or without treatment with the indicated concentrations of clustered DLL1 or monovalent soluble JAG1 constructs. Expression of CD62L and CD44 was assessed on gated CD8 population as indicated by flow cytometry. Dot plots from a representative experiment out of two independent experiments with duplicates are shown. (PPTX 4553 kb)

Additional file 2: **Figure S2.** Clustered DLL1 and soluble JAG1 constructs decrease the expression of checkpoint molecule PD-1 on T-effector memory cells in vitro. Purified T cells were stimulated in vitro in a T:DC (3:1) stimulation co-culture with allogenic dendritic cells in the presence of CD3/CD28 beads (1 μg/mL) for four days with or without treatment with the indicated concentrations of clustered DLL1 or monovalent soluble JAG1 constructs. Expression of CTLA-4 and PD-1 was assessed on gated populations as indicated by flow cytometry. Dot plots from a representative experiment out of two independent experiments with duplicates are shown. (PPTX 8407 kb)

Additional file 3: **Figure S3.** T-cell expressed PD-1 and NOTCH receptors correlate with DC-expressed NOTCH ligands in human lung tumor-infiltrate. Heatmap shows Pearson’s correlation between the indicated populations. *P*-values were corrected by Benjamani-Hochberg procedure. Color code indicates the strength of correlation and direction; * *p* < 0.05. (PPTX 181 kb)

## Abbreviations

APC: Antigen presenting cells
DC: Dendritic cells
DLL: Delta-like Notch ligands
Jag: Jagged
LLC: Lewis Lung Carcinoma
MLR: Mixed lymphocyte reaction
