# Correction to: Determinant roles of dendritic cell-expressed Notch Delta-like and Jagged ligands on anti-tumor T-cell immunity

**DOI:** 10.1186/s40425-019-0592-2

**Published:** 2019-05-07

**Authors:** Elena E. Tchekneva, Mounika U. L. Goruganthu, Roman V. Uzhachenko, Portia L. Thomas, Anneliese Antonucci, Irina Chekneva, Michael Koenig, Longzhu Piao, Anwari Akhter, Maria Teresa P. de Aquino, Parvathi Ranganathan, Nicholas Long, Thomas Magliery, Anna Valujskikh, Jason V. Evans, Rajeswara R. Arasada, Pierre P. Massion, David P. Carbone, Anil Shanker, Mikhail M. Dikov

**Affiliations:** 1 Division of Medical Oncology, Department of Internal Medicine, The Ohio State University Wexner Medical Center and The James Comprehensive Cancer Center, 460 W 12th Ave, 484 BRT, Columbus, OH 43210 USA; 2 Department of Biochemistry, Cancer Biology, Neuroscience and Pharmacology, Meharry Medical College School of Medicine, 2005 Harold D. West Basic Sciences Building, 1023 21st Ave N, Nashville, TN 37208 USA; 3 Department of Microbiology, Immunology and Physiology, Meharry Medical College School of Medicine, Nashville, USA; 4 School of Graduate Studies and Research, Meharry Medical College, Nashville, TN USA; 5 Sechenov First Moscow State Medical University, Moscow, Russia; 6 Division of Hematology, Department of Internal Medicine, The Ohio State University Wexner Medical Center, Columbus, OH USA; 7 Department of Chemistry and Biochemistry, The Ohio State University, Columbus, OH USA; 8 Department of Inflammation and Immunity, Cleveland Clinic, Cleveland, OH USA; 9 Department of Pathology, West Virginia University, Morgantown, WV USA; 10 Department of Medicine, Vanderbilt University, Nashville, TN USA; 11 Host–Tumor Interactions Research Program, Vanderbilt-Ingram Comprehensive Cancer Center, Vanderbilt University, Nashville, TN USA; 12 Vanderbilt Institute for Infection, Immunology and Inflammation, Vanderbilt University, Nashville, TN USA

**Correction to: J Immunother (2019) 7:95**

**https://doi.org/10.1186/s40425-019-0566-4**

Following publication of the original article [1], the author reported the wrong version of Figs. 5 and 7 have been published. The correct version of the figures can be found below:

**Fig. 5:**
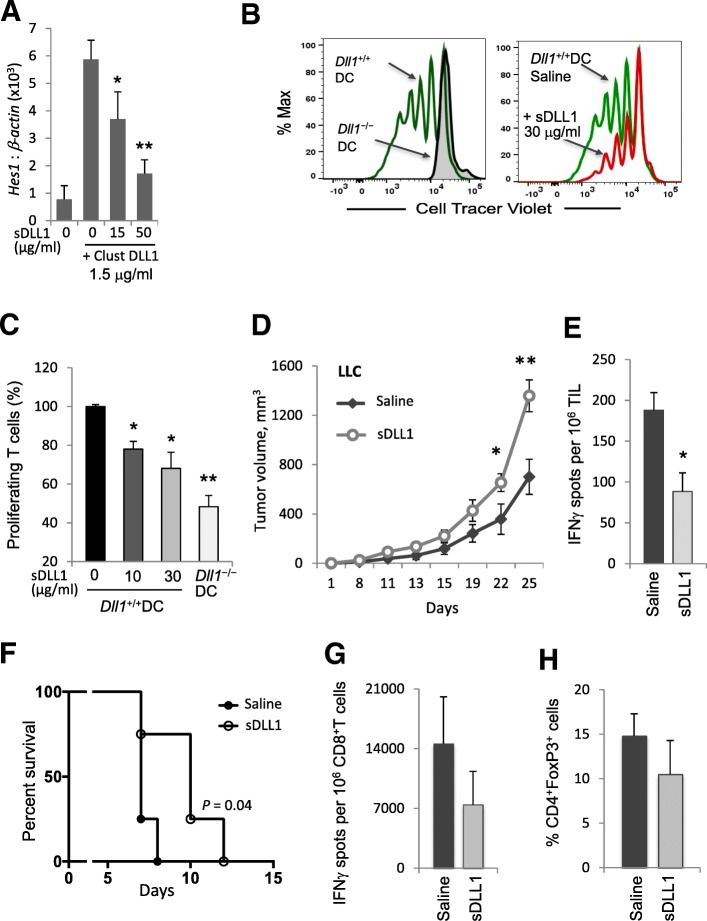
Monomeric soluble DLL1 or *Dll1*-ablated dendritic cells restrict Notch signaling and impair T-cell cytotoxic responses. **a** Expression of Notch downstream target *Hes1* mRNA was assessed by qRT-PCR in 3 T3 cells treated with clustered DLL1 in the presence of soluble DLL1 (sDLL1) construct at indicated concentrations for 16 h. **b**, **c** T-cell proliferation was measured after co-incubating allogeneic T-cells labeled with Cell Tracer Violet fluorescent dye with bone marrow-derived *Dll1*^*−/−*^ or wild-type DC in the presence of soluble anti-CD3 for 5 days. In some T-cell cultures with wild-type DC, soluble DLL1 construct was added at the indicated concentrations. Representative Cell Tracer Violet dye dilution profile is shown (**b**). **d** Tumor volume was measured in LLC tumor-bearing mice treated with sDLL1 construct 1 mg/kg body weight, i.p. every 2 days for 20 days. **e** IFN-γ producing tumor-infiltrating cells from these mice were enumerated by ELISPOT assay on day 18 after LLC tumor initiation. Mean ± SEM, 8 mice per group; *, *p* < 0.05; **, *p* < 0.005. **f**, **g** C57BL/6 mice were transplanted with BALB/c heart allografts on day 0 and treated with sDLL1 construct (1 mg/kg) i.p. on days − 3, − 1, 1, 3, 5 and 7. **f** Heart allografted C57BL/6 mice log-rank survival. **g** IFN-γ ELISPOT assay on recipient CD8^+^T cells isolated after heart allograft and re-stimulated with mitomycin C-treated donor spleen cells in the presence of recipient C57BL/6 splenocytes. **h** Percentage of FoxP3^+^ cells among CD4^+^ splenocytes after heart allograft. Mean ± SEM, 4–8 mice per group; *, *p* < 0.05

**Fig. 7:**
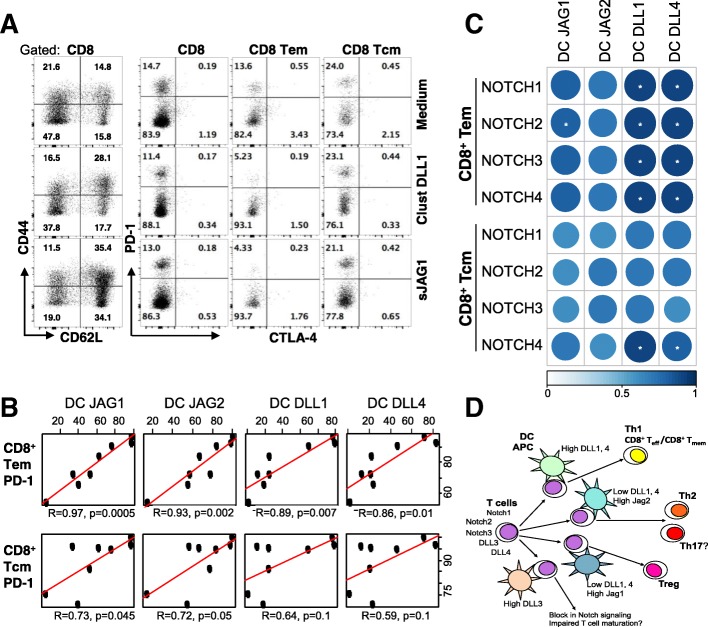
Dendritic cell Jagged expression correlates with PD-1 expression on T-effector-memory cells. **a** Purified T cells were stimulated in vitro in a T:DC (3:1) stimulation co-culture with allogeneic bone marrow-derived dendritic cells in the presence of CD3/CD28 beads (1 μg/mL) for four days with or without treatment with clustered DLL1 (1.5 μg/mL) or monovalent soluble JAG1 (20 μg/mL) constructs. Expression of CD62L, CD44, CTLA-4 and PD-1 was assessed on gated populations as indicated by flow cytometry. Dot plots from a representative experiment out of two independent experiments with duplicates are shown. **b**-**c** Lung tumor single cell suspensions from 10 patients were evaluated for the expression of NOTCH ligands on tissue-resident CD11b^+^CD11c^high^ dendritic cells and PD-1 and NOTCH receptors in populations of T cells by flow cytometry. NOTCH ligands in CD11b^+^CD11c^high^ cells were compared to PD-1 positivity of Tem and Tcm cells (**b**) or to NOTCH receptor positive T cell subsets by Pearson’s correlation (**c**). All *p*-values were corrected using the Benjamani- Hochberg procedure; *n* = 8; * *p* < 0.05. Color code indicates the strength of correlation. **d** Scheme summarizing available data on the regulation of T cell responses by Notch ligands

The original article has been corrected as well.
