# Supplementary figures and images for: Determinant roles of dendritic cell-expressed Notch Delta-like and Jagged ligands on anti-tumor T cell immunity
Source: J Immunother Cancer. 2019 Apr 2;7:95. doi: 10.1186/s40425-019-0566-4 (PMC6446314; doi:10.1186/s40425-019-0566-4)

## Slide 1
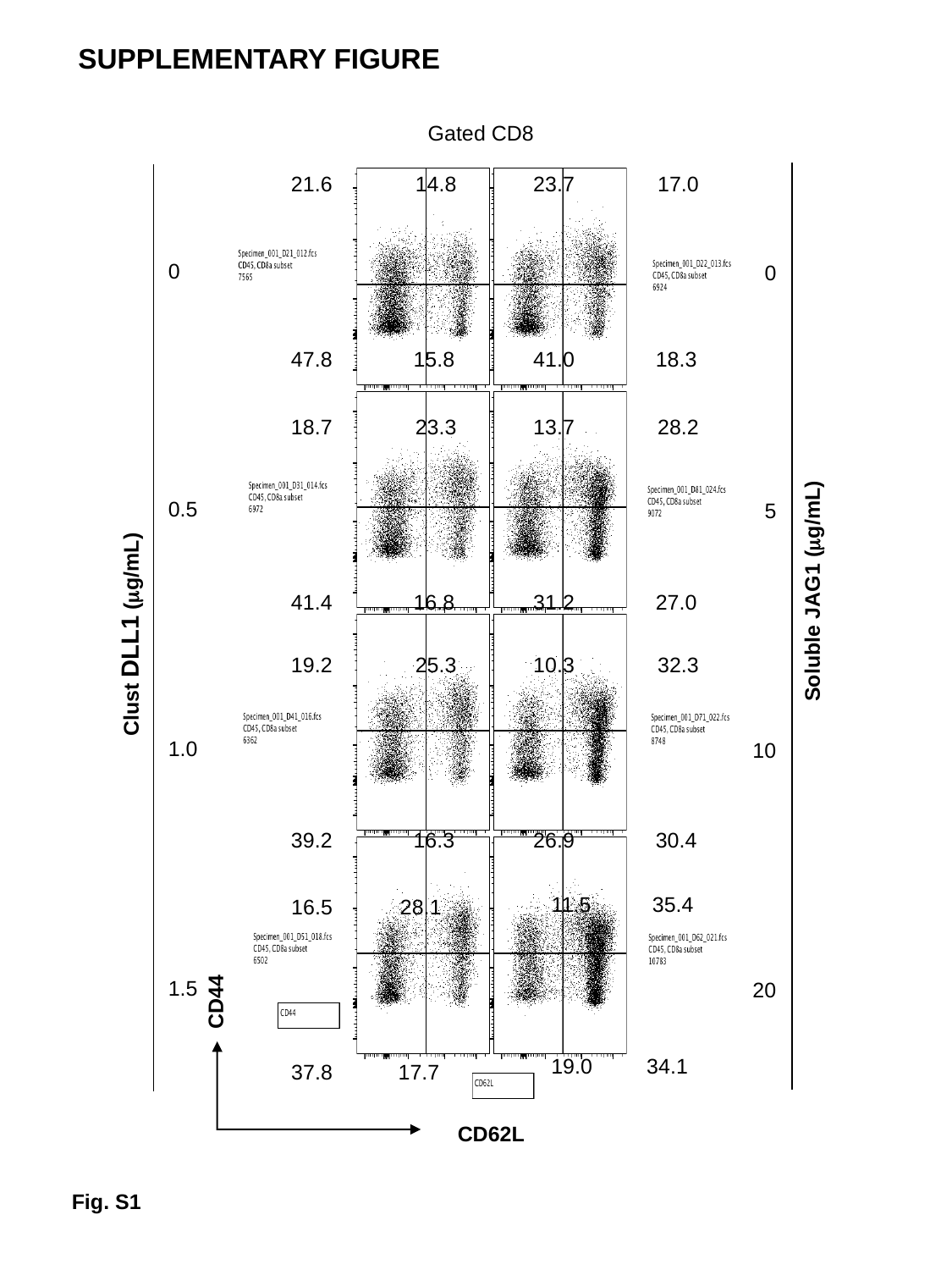

SUPPLEMENTARY FIGURE
Gated CD8
21.6
14.8
23.7
17.0
0
0
47.8
15.8
41.0
18.3
18.7
23.3
13.7
28.2
 Soluble JAG1 (mg/mL)
0.5
5
 Clust DLL1 (mg/mL)
41.4
16.8
31.2
27.0
19.2
25.3
10.3
32.3
1.0
10
39.2
16.3
26.9
30.4
11.5
35.4
16.5
28.1
1.5
20
CD44
19.0
34.1
37.8
17.7
CD62L
Fig. S1

Supplement: Supplementary file 1 — Figure S1. Clustered DLL1 and soluble JAG1 constructs modulate the differentiation of memory T-cells in vitro. Purified T cells were stimulated in vitro in a T:DC (3:1) stimulation co-culture with allogeneic bone marrow-derived dendritic cells in the presence of CD3/CD28 beads (1 μg/mL) for four days with or without treatment with the indicated concentrations of clustered DLL1 or monovalent soluble JAG1 constructs. Expression of CD62L and CD44 was assessed on gated CD8 population as indicated by flow cytometry. Dot plots from a representative experiment out of two independent experiments with duplicates are shown. (PPTX 4553 kb) [file 40425_2019_566_MOESM1_ESM.pptx]

## Slide 1
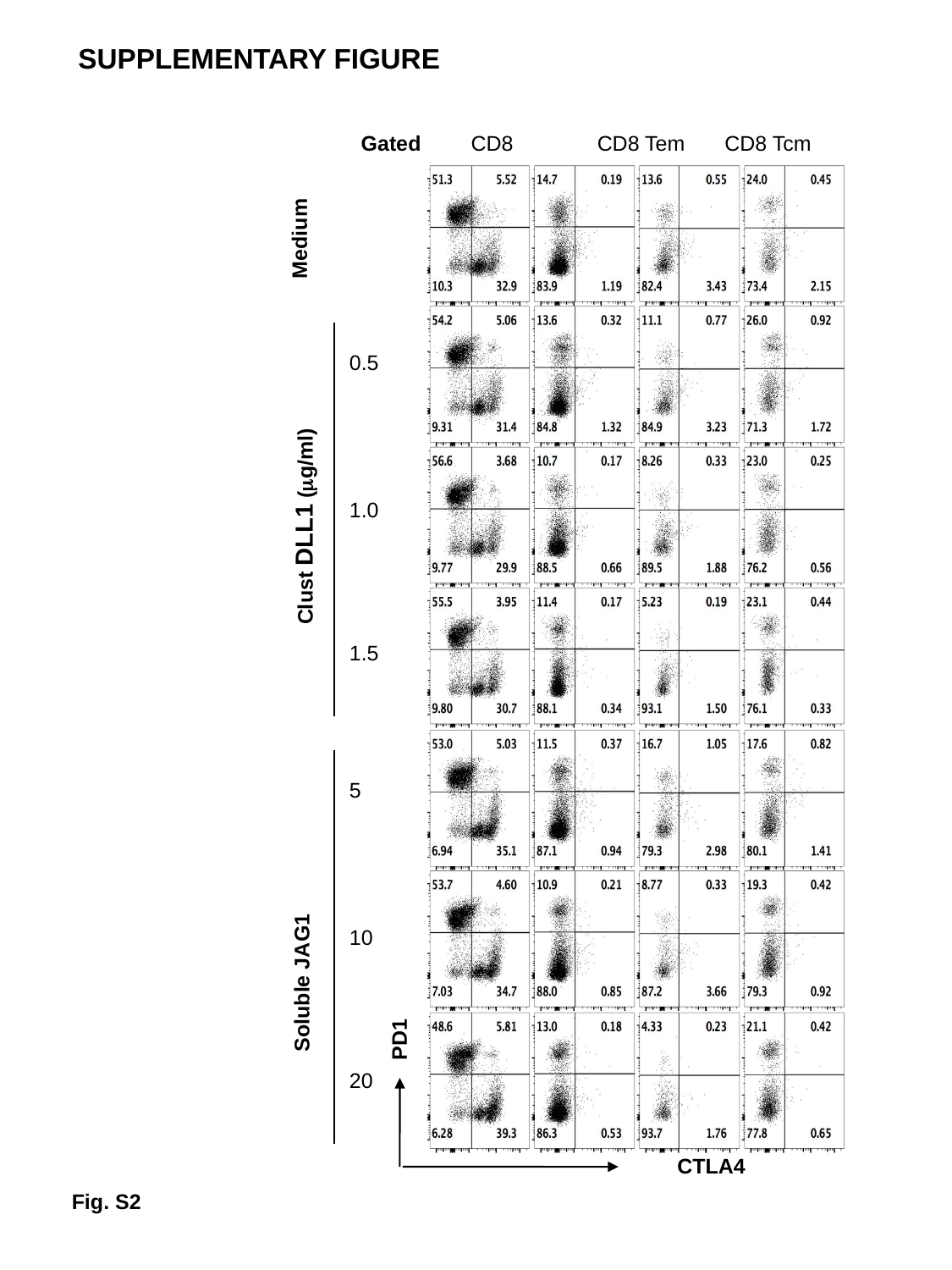

SUPPLEMENTARY FIGURE
Gated
CD8
CD8 Tem
CD8 Tcm
Medium
0.5
 Clust DLL1 (mg/ml)
1.0
1.5
5
 Soluble JAG1
10
PD1
20
CTLA4
Fig. S2

Supplement: Supplementary file 2 — Figure S2. Clustered DLL1 and soluble JAG1 constructs decrease the expression of checkpoint molecule PD-1 on T-effector memory cells in vitro. Purified T cells were stimulated in vitro in a T:DC (3:1) stimulation co-culture with allogenic dendritic cells in the presence of CD3/CD28 beads (1 μg/mL) for four days with or without treatment with the indicated concentrations of clustered DLL1 or monovalent soluble JAG1 constructs. Expression of CTLA-4 and PD-1 was assessed on gated populations as indicated by flow cytometry. Dot plots from a representative experiment out of two independent experiments with duplicates are shown. (PPTX 8407 kb) [file 40425_2019_566_MOESM2_ESM.pptx]

## Slide 1
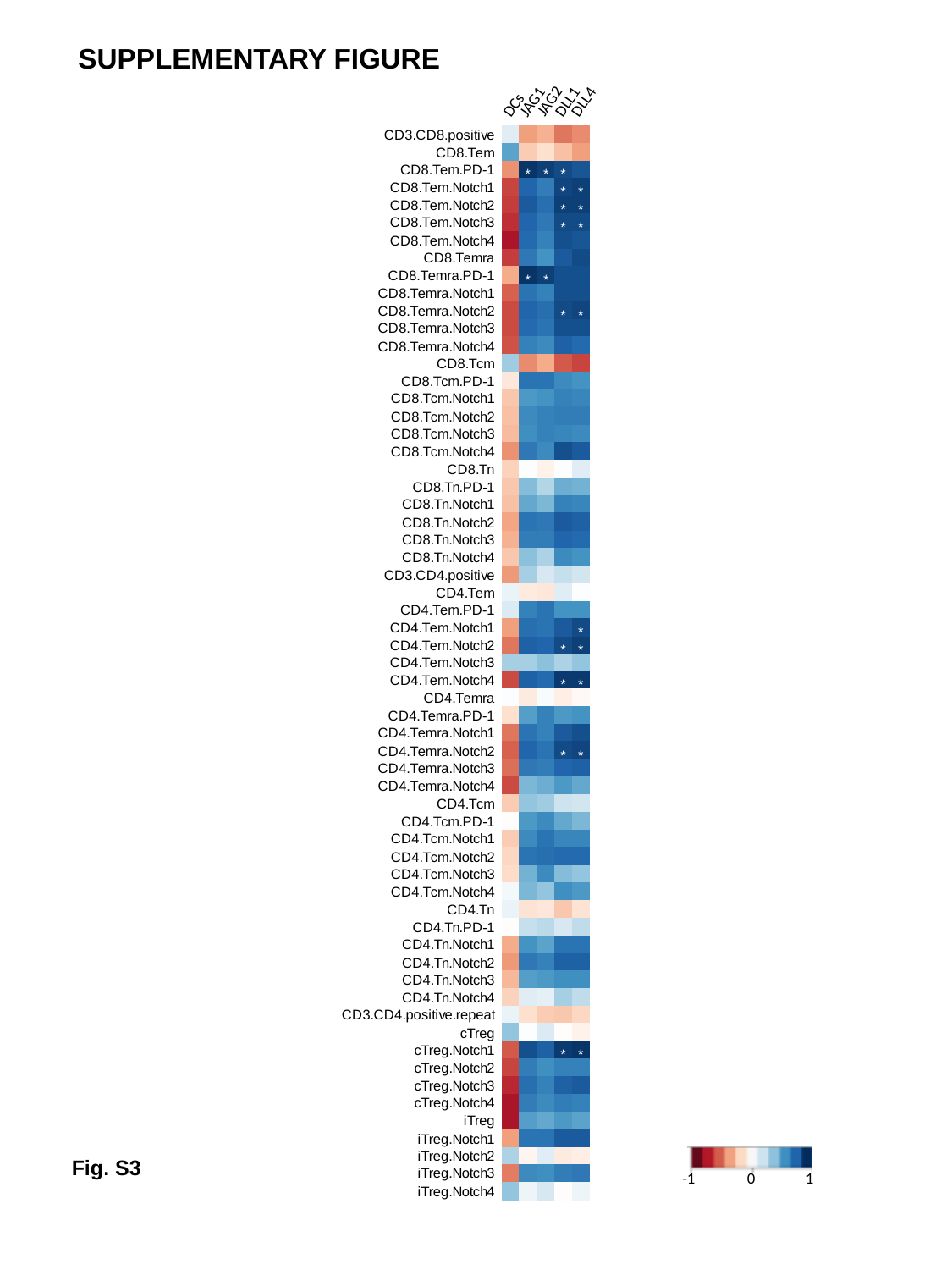

SUPPLEMENTARY FIGURE
JAG2
DLL4
DLL1
JAG1
DCs
-1
0
1
Fig. S3

Supplement: Supplementary file 3 — Figure S3. T-cell expressed PD-1 and NOTCH receptors correlate with DC-expressed NOTCH ligands in human lung tumor-infiltrate. Heatmap shows Pearson’s correlation between the indicated populations. P-values were corrected by Benjamani-Hochberg procedure. Color code indicates the strength of correlation and direction; * p < 0.05. (PPTX 181 kb) [file 40425_2019_566_MOESM3_ESM.pptx]
